# Sociodemographic structure and health care-related outcomes of community-dwelling dementia caregiving dyads: a latent class replication study

**DOI:** 10.1186/s12913-023-09505-5

**Published:** 2023-05-12

**Authors:** Henrik Wiegelmann, Karin Wolf-Ostermann, Niels Janssen, Hein van Hout, Janet L. MacNeil Vroomen, Farhad Arzideh

**Affiliations:** 1 Institute for Public Health and Nursing Research, University of Bremen, Bremen, Germany; 2 Department of Psychiatry and Neuropsychology, School for Mental Health and Neuroscience, Alzheimer Centre Limburg, Maastricht University, Maastricht, the Netherlands; 3 General Practice & Medicine of Older People, Amsterdam University Medical Center, Amsterdam, the Netherlands; 4 Internal Medicine, Section Geriatrics, Amsterdam University Medical Center, Amsterdam, the Netherlands; 5 University Medical Center Knappschaftskrankenhaus Bochum, Bochum, Germany

**Keywords:** Dementia, Informal care, Dementia dyads, Psychosocial health, Health care service use, Quality of life, Caregiver burden, Latent class analysis, Replication study

## Abstract

**Background:**

The identification of dyadic subgroups of individuals living with dementia and their informal caregivers can help to design effective tailored support. In a previous German study, we identified six dementia dyad subgroups by applying Latent Class Analysis (LCA). Results showed sociodemographic heterogeneity as well as differences in health care outcomes (i.e., quality of life, health status, caregiver burden) between subgroups. The objective of this study is to determine if the dyad subgroups from the previous analysis can be replicated in a similar but distinct Dutch sample.

**Methods:**

A LCA 3-step procedure was applied to baseline data from the COMPAS study, a prospective cohort study. LCA is a statistical approach used to identify heterogeneous subgroups within populations based on their pattern of answers on a set of categorical variables. Data comprises 509 community-living individuals with predominantly mild to moderate dementia and their informal caregivers. A narrative analysis was used to compare latent class structures of the original versus the replication study.

**Results:**

Six distinct dementia dyad subgroups were identified: A subgroup of “adult–child-parent relation with younger informal caregiver” (31.8%), a “couple with female informal caregiver of older age” group (23.1%), an “adult–child-parent relation with middle-aged informal caregiver” group (14.2%), a “couple with middle-aged female informal caregiver” group (12.4%), a “couple with older male informal caregiver” group (11.2%) and a “couple with middle-aged male informal caregiver” group (7.4%). Quality of life of individuals with dementia was rated better in couples than in adult–child-relationships. Worst health for caregivers was reported by subgroups with female informal caregivers living together with male individuals with dementia in couple relationships. A subgroup with older female informal caregivers in couple relationships report the most severe burden on physical and mental health. In both studies, a model with six subgroups fitted the data best. Although substantive similarities between the subgroups of both studies appeared, considerable differences are also evident.

**Conclusion:**

This replication study confirmed the existence of informal dementia dyad subgroups. The observed differences between the subgroups provide useful contributions for a more tailored health care services for informal caregivers and individuals living with dementia. Furthermore, it underlines the relevance of dyadic perspectives. To facilitate replication studies and increase the validity of evidence, a standardization of collected data across studies would be beneficial.

**Supplementary Information:**

The online version contains supplementary material available at 10.1186/s12913-023-09505-5.

## Background

Dementia is a global public health and social care challenge affecting various societal levels ranging from individuals, families, communities to governments [1, 2]. Dementia is characterized with deterioration in memory, thinking, behavior and the ability to perform everyday activities [3]. Looking back at the past 30 years, there has been a global increase in the total numbers of individuals living with dementia. This is mainly driven by increasing life expectancy and an accompanying increase in the number of elderly people over 80 years of age when dementia is most prevalent [4]. It is expected that the total number of individuals living with dementia will continue to increase globally up to 152.8 million in 2050 [5].

This continued increase in the absolute numbers of cases means that the number of professional caregivers and especially informal caregivers that will be needed to support growing numbers of individuals living with dementia will also increase simultaneously [6]. Many studies indicated that informal caregivers of individuals living with dementia have an increased risk of physical and psychosocial health problems resulting from the multiplicity of care tasks they fulfil. As a further consequence, they may face social and economic disadvantages, which could impact the care quality and the situation of the care recipient [7–10]. This is not to negate the fact that providing informal care is also accompanied by positive experiences like satisfaction, rewards, enjoyment, or personal growth [11, 12]. However, the large number of studies on unmet needs of informal caregivers of individuals living with dementia shows that, despite the variety of existing care services, there is still a great need for improved and targeted support structures [13]. This needs-based tailored support is important since otherwise not only informal caregiver’s quality of life (QoL) is affected but also crises (i.e., unwanted institutionalization) may develop among individuals with dementia and informal caregivers [14]. Since individuals living with dementia and their informal caregivers are not a homogenous group but have distinct individual needs, tailoring health care services to the needs of specific dyadic target groups could help make support more efficient. This is also indicated by previous research [15].

Several studies in psychosocial care and dementia research have used latent class approaches to address issues of informal caregiving [16–21]. Beeber et al. [16] as well as Janssen et al. [17] examined patterns of health and social care service use. Their studies emphasize the relevance of specific characteristics (i.e., diagnosis of mild cognitive impairment (MCI) or dementia, impairment in activities of daily living, age of care recipient and living situation) of subgroups for the planning of tailored support strategies. Janssen et al. [18] identified five dementia caregiver profile types with significant differences regarding the psychosocial outcome’s quality of life, depressive symptoms and perseverance time. Pristavec [19] applied LCA to a U.S. data set and identified five different informal caregiving experiences classes with distinct benefit and burden levels associated with medical, caregiving and sociodemographic aspects. Yuan and colleagues [20] discovered three classes of informal caregivers of individuals living with dementia, with different coping patterns and its impact on caregivers. The classes differed regarding i.e., caregivers’ personal characteristics and caregiving stressors (behavior of individuals with dementia, caregiving burden). The study by Jutkowitz et al. [21] focuses on profiles of dementia caregiving arrangements and revealed three distinct classes of caregiving networks with. Depending on the class, different actors play the dominant network role (i.e., children, paid care, spouse), something to be considered when addressing the caregiving networks with support services.

Hence, there are several LCA studies in the field of psychosocial dementia and care research, which gives an illustration of the wide range of possible applications for LCA. However, to the best of our knowledge, LCA that explore different dementia care dyad profiles based on socio-economic aspects are not available, except for the original study to be replicated here. Therefore, this study is of relevance because it replicates a previous study approach as closely as possible with a new study sample and thus provides further verification of the initial findings.

In this previous study [22] a LCA was performed on baseline data of a German sample comprising 551 individuals living with dementia and their informal caregivers. Six distinctive dementia care dyad subgroups were found and were labelled as, (1) “adult child parent relationship with younger informal caregiver”, (2) “adult child parent relationship with middle-aged informal caregiver”, (3) “nonfamily relationship with younger informal caregiver”, (4) “couple with male informal caregiver of older age”, (5) “couple with female informal caregiver of older age”, (6) “couple with younger informal caregiver”. These six subgroups furthermore showed to differ significantly about individual, relational and social aspects. Results highlighted the need to approach dyad subgroups specifically in terms of promoting health, easing of burden and improvement of quality of life, i.e., through tailored counselling, promotion of existing services and development of target group-specific support services.

This replication study is based on a secondary analysis of Dutch data. In the Netherlands 290.000 individuals are living with dementia, of which approx. 79% live in the community, a situation that is often wanted and chosen by the individuals and relatives themselves but is also politically preferred and supported accordingly [23, 24]. These ratios are quite similar in Germany, with a slightly higher overall dementia incidence. In relation to the population size, the overall economic costs associated with dementia care in the Netherlands are immense, largely driven by the high utilization of formal care [25, 26]. The estimated number of informal caregivers of individuals with dementia in the Netherlands is approximately 350.000. For Germany, it can be assumed that there are approximately 1.7 million informal dementia caregivers over the age of 40, based on own calculations using data from the German Aging Survey. The overall sociodemographic characteristics of the Dutch dementia caregiver population are similar to those of other Western European countries, including Germany, with an average age of 65 years and a share of female caregivers of 68%. Table 1 shows key information on the population under study in comparison between the Netherlands and Germany.

**Table 1:** The Netherlands and Germany: Comparison of data on dementia population and informal caregivers

	**Netherlands**	**Germany**
Individuals with dementia (total number estimated)	290.000 [23]	1.6 m [27]
Individuals with dementia per 1000 population (own calculations)	16.1	20.2
Individuals with dementia living (and cared for) at home	79% [23]	75% [28]
Informal caregivers of individuals with dementia	350.000 [23]	1.7 m [29]
Mean age informal caregiver of individuals with dementia	65.0 [30]	60.5 [27]
Female informal caregivers of individuals with dementia	68% [30]	75% [27]
Dementia costs per year (2015)	6.6bn [26]	18bn [28]

### Research questions and aims

This study addresses the following three research questions: Which latent classes of informal dementia care dyads can be identified in the Dutch COMPAS dataset using the same methodological and statistical approach of the original German study? What are the similarities and differences regarding health care related outcomes (i.e., quality of life, burden of care, unmet needs) between the Dutch latent classes? What are the similarities and differences when comparing the latent classes of the German original study and the present Dutch replication study?

Derived from these research questions, the study pursues the overarching aim to determine whether the latent class structure of community-dwelling informal dementia care dyads, characterized in a previous German data set, replicates in a structurally similar but distinct Dutch sample of individuals living with dementia and their informal caregivers. The analysis has the following three specific aims:

- a) identification of latent classes in the Dutch COMPAS dataset using the methodological and statistical approach of the original German study [22],
- b) analysis of correlations with distal outcomes (i.e., quality of life, burden of care, unmet needs) in COMPAS data set using the LCA 3-step approach,
- c) comparison of results of the current study and the previous study.

The results will show whether findings are likely to be generalizable, especially since the sample in this study is drawn from a different population. This might build up evidence (greater external validity), on which to base better tailored health care support services for community-dwelling dementia care dyads [31].

## Methods

### Study design

The present study is designed as a replication of a previous LCA, which was carried out with baseline data of the German DemNet-D study [22]. Replication studies strive to duplicate a certain research approach in a second investigation. Replication studies can help to get a better picture of the generalizability of initial results, especially when results are based on a different population sample. Overall, studies of this type contribute to building up evidence, on which improved health care supply structures can then be designed and implemented [31, 32]. Since there are no specific standards for replication studies, we followed the STROBE (Strengthening the Reporting of Observational Studies in Epidemiology) guidelines [33] (Additional file 1).

### Setting and participants

The original study to which we refer in this replication approach is the DemNet-D study. This is a multidimensional and multidisciplinary longitudinal evaluation study (2012–2015), which investigated the care and living situation of community-dwelling individuals living with dementia and their informal caregivers as service users of thirteen regional dementia networks (DCN) in different regions of Germany. The DemNet-D LCA included baseline data from 551 individuals with dementia and their 551 informal caregivers. Individuals with dementia could take part if they lived at home and had dementia (formally diagnosed by a medical professional or as reported by the informal caregiver). In addition, they had to have an informal caregiver and be registered as service users in one of the 13 dementia care networks that were practice partners in the DemNet-D study. Informal caregivers were eligible if they were primary caregivers of an individual with dementia and if they were able to provide detailed information on the individual with dementia. Furthermore, they had to live preferably in the same household with the individual with dementia. The original study design and the population included have already been published more detailed in several publications [34, 35].

For the present study, we set the goal to replicate the methodical and statistical approach of the DemNet-D LCA with a different study sample from a different country. We used the structurally and thematically similar Dutch COMPAS dataset, a mixed-method prospective, observational and controlled cohort study conducted in the Netherlands. The main aim of the COMPAS study was to evaluate the effects of two case management models compared to care in regions where patients had no access to case management [24]. Dementia dyads were recruited from both urban and rural regions. Individuals living with dementia were eligible for this study if they lived at home, had a formal diagnosis of dementia, were not terminally ill, were not anticipated to be admitted to a long-term care facility within six months, and had an informal caregiver. The informal caregivers were eligible if they were primarily responsible for looking after the person with dementia, had sufficient language proficiency, and were not severely ill. Detailed descriptions of the methodological approach have already been published earlier in several studies [36–38]. For this study, we used cross-sectional baseline data (*n* = 509), including both individuals with and without case management service. The original COMPAS data set includes 521 dyads. Twelve dyads were removed because data was missing on at least half of the indicator variables used for identifying the classes. As much as possible, we matched the measures used in the COMPAS study with measures used in the original study [27]. A direct comparison of all indicator variables used in both studies can be taken from supplementary table 1 (Additional file 2).

### Indicator variables

In LCA, indicator variables are dependent variables used to determine the latent classes [39]. For this current study, we tried to model all indicators as closely as possible to the original indicators or included proxy indicators. The coding details of all indicator variables can be taken from supplementary table 1 (Additional file 2). Indicators related to the individuals with dementia, the informal caregiver, the dyad level and the region level are detailed below.

### Indicators for individuals living with dementia

Sociodemographic indicators include age, sex, and education of the person with dementia. Activities of daily living using the modified Katz-15 ADL index [40] were dichotomized into higher functioning and less dependent (0–4) versus lower functioning and more dependent (5–15). The presence of dementia-related behavioral symptoms was determined with presence or absence of “agitation”, “aggression”, and “inappropriateness” using the Neuropsychiatric Inventory (NPI) [41]. These three items were selected because they were found to make significant contributions to the latent class structure identified in the original study using the Cohen-Mansfield Agitation Inventory (CMAI) [42]. Cognitive impairment was measured with the Mini Mental Status Examination (MMSE) [43], ranging between 0–30. For the LCA, we dichotomized the MMSE into no to mild cognitive impairment (30–21) and moderate to severe cognitive impairment (20–0) [44].

### Indicators for informal caregivers

The sociodemographic data we included for the informal caregivers were age, sex, and whether they are in paid employment. The amount of weekly care and support given was assessed using summed values from three self-composed and piloted single items from which a graded classification of low (0–14 h/week), moderate-high (15–56 h/week) and very high (57 h and more/week) was created [45]. To estimate the duration of care (in months), we included a proxy question for the informal caregivers about when the dementia symptoms started. This indicator has been coded as a binary variable, using the mean value of 50 months as the cut-off point (up to 50 months/more than 50 months).

### Indicators used at the dyadic level

At the dyadic level, four indicators were included. First, we accounted for the informal care relationship and differentiated between couple, adult–child and other/non-kinship relationships. Second, information on whether the person with dementia and the informal caregiver live together and third, whether other (informal) support is involved in the dementia care arrangement was included. To account for the socio-economic situation of the dyad we used the education of the person living with dementia (graduation) as a proxy measure, which we have classified into low, medium, and high education.

### Indicators used at the regional level

For the consideration of structural social inequalities, we included a measure on the regional socio-economic status (RSES) at postal code level and formed three groups (lower RSES, middle RSES, upper RSES) based on terciles [46].

### Distal outcomes

In LCA, distal outcomes can be introduced to investigate the effect of membership in latent classes on an external variable of interest. All distal outcomes described below were rated by the informal caregivers at baseline, and detailed information can be taken from supplementary table 2 (Additional file 2).

### Health care service use

The utilization of professional health care services was assessed by using several single items from the COMPAS questionnaires (37, 38) These were grouped into the categories of medical, therapeutic, and nursing services. In each case, it was assessed whether services in this domain have been used or not. To determine the use of resources used by participants to gather information on health care issues also single items from the COMPAS study were used. Again, we grouped individual questions into three major domains, medical, nursing and civil society resources. In each case, it was assessed whether information in those domains have been used or not. As in the original study, we did not include case management itself as a direct health care service in the analyses, given its predominant coordinating role in the provision of health care services.

### Quality of life for individuals living with dementia

To determine the quality of life of the individuals living with dementia, the Quality of Life-Alzheimer’s Disease (QoL-AD) instrument was used as a proxy measure. The total score of the QoL-AD ranges between 13–52. A higher value indicates a better QoL [47].

### Mental health and caregiver burden for informal caregivers

The mental health of informal caregivers was assessed using the 12-Item Short-Form Health Survey (GHQ-12). This tool consists of twelve statements that respondents can rate on a four-point Likert-type scale (0 = Not at all; 3 = More than usual). The score was used to generate a total score between 0–36, where higher scores indicate worse health [48]. Furthermore, the CarerQoL instrument was included, which aims to measure care-related quality of life in informal caregivers. For this study, the 7-item scale that evaluates relevant care burden dimensions was used and items (scoring: 0 = no, 1 = some, 2 = a lot) were analyzed separately [49].

### Unmet needs for home-based care arrangement

The CANE (Camberwell Assessment of Need for the Elderly) was applied as a proxy measure to assess the stability of home-based care arrangements for the person with dementia [50]. The tool can be used to determine the number of unmet needs on a scale ranging between 0–26.

### Statistical analysis

The statistical package Latent Gold 6.0 was used for all analyses [51]. To identify meaningful subgroups of dementia care dyads as well as their effects on healthcare-related outcomes, a bias-adjusted Step-3 LCA model with distal outcomes was applied. All methodological and statistical procedures were applied as in the original study [22].

LCA is a person-centered and probabilistic statistical approach for categorical data. It belongs to the group of finite mixture models (FMM) which assume that there are two or more distinct groups hidden in a heterogeneous population [52]. Individual membership to these groups is based on response patterns to a set of observable items, or indicator variables. On the one hand, members of one group have maximally similar response patterns (class homogeneity) but on the other hand, those patterns differ maximally compared to members of other groups (class separation) [53, 54].

The 3-Step approach was used because the interest of the current study was not only to identify different types of dementia care dyads, but also in relating the membership of these types to distal outcomes (healthcare-related outcomes) of interest [55]. The 3-Step approach includes the following stages:

1. *Step:* In a first step, a best-fit latent class model is established for a set of indicator variables.
2. *Step:* The second step comprises the probabilistic assignment of single cases to the latent classes.
3. *Step:* The third step uses the probabilistic scores and examines the association between latent class membership and the distal outcomes chosen. To prevent bias, the associations are corrected for the classification error [55].

Subsequently, the results of both studies were compared in a narrative way. This allows a specific assessment of similarities and differences between the two LCA.

### Procedure of latent class analysis

In a first step, we estimated the number of classes, class sizes and class structure. A hierarchical list (see Table 2) of indicator variables formed the basis for this. The hierarchy was developed based on previous research findings [56] as well as the study aims set. Variables were added gradually to the model calculations. The first indicator variables (level 1) were used to determine the optimized number of classes and hereby the main characteristics of the classes. This number of classes (*n* = 6) was set despite adding further indicator variables of levels 2–4. Following this procedure, the optimized 6-class model based on primary indicators remained, and further indicators only influenced the class characteristics (probabilities) if they were significant. To reduce the probability of local optima, we used the integrated option in Latent Gold software and repeated the algorithm with different starting points chosen at random.

**Table 2:** Hierarchy of indicator variables for replication LCA with COMPAS data

Level	Indicator variable
1	
	Age of informal caregivers
	Age of individuals living with dementia
	Informal care relationship
	Sex of informal caregivers
	Sex of individuals living with dementia
	Living situation of dyad
2	
	Further informal support
	Paid work of informal caregivers
	Education of individuals living with dementia
	Time informal caregivers spent for care and support
	Start of dementia symptoms
3	
	Activities of daily living (Katz ADL-15)
	Neuropsychiatric Inventory (NPI) – 3 Items
	Mini Mental Status Examination (MMSE)
4	
	Regional socio-economic status (RSES)

The inclusion of lower ranking variables (level 2–4) was made after all higher-ranking variables had been tested. A first LCA was conducted using the six variables of level 1: sex and age of informal caregivers, sex and age of person with dementia, informal care relationship and the living situation of the dyad. Different model solutions were evaluated using the Bayesian information criterion (BIC), the entropy score as well as interpretation by the authors involved. To test whether the influence of individual indicator variables on the model is significant, we used the Wald-Test and the Likelihood Ratio Test (LRT). Both tests were applied to assess if the regression coefficients within all classes are equal to zero (null hypothesis), and whether the model with six indicator variables represents a significant improvement compared to models with fewer variables (null hypothesis test). The variables of level 2–4 were added one by one to the model afterward. If a variable did not significantly improve the fit of the overall latent class model, it was excluded. Finally, BIC and entropy were examined to test if the final model with six classes and all variables sufficiently represented the data. The Expectation–Maximization (EM) algorithm was applied for the maximum likelihood (ML) estimation of the model. The EM algorithm uses no imputation algorithm for missing values. The only assumption is that missing data is missing at random (MAR). We used all observed attributes for each individual case.

## Results

### Sample characteristics

A total of 509 community-dwelling informal dementia care dyads were included in this study. The sociodemographic and clinical details of this population are summarized and compared with sample characteristics of the original German LCA in supplementary table 3 (Additional file 2). In the current study, individuals living with dementia were on average 79.7 years old (SD: 7.9) of which more than half were female (55%). The majority (54.6%) of individuals with dementia had a mid-level education (secondary school), about every fifth (21.8%) had a lower educational level (6 or fewer classes) and an only slightly smaller group (18.3%) a high-level education (minimum higher secondary education). Despite the formal dementia diagnosis, which was a requirement for participation in the study, 42.2% of the individuals with dementia showed no or only mild cognitive impairments according to the administered MMSE. However, it is known that individuals with a dementia diagnosis may perform proportionately well on the MMSE than a diagnosis may suggest [57]. 28.3% showed moderate or severe cognitive deficits. For 29.5% there was no data on MMSE testing available, as one of the participating case management organizations asked not to burden their clients additionally with the MMSE. Based on the KATZ ADL-15 scale, almost three-quarters (73.1%) of the individuals with dementia reported limitations in five or more domains. Scoring on the NPI showed that inappropriate behavior was present in 31.4%, aggressive behavior in 40.1% and agitated behavior in 41.7% of the individuals living with dementia. Quality of life (QoL-AD) of the individuals living with dementia, as reported by informal caregivers, showed a mean of 30.3 (SD: 5.8).

The mean age for informal caregiver was 64.5 (SD:12.5) and two-third were female (66.6%). In addition to their care responsibilities, 38.9% were also in paid work. The time spent on care and support varies considerably. Almost half of the informal caregivers (48.9%) have spent between 0–14 h per week (h/week), 27.5% spent a moderate to high amount (15–56 h/week), and 6.9% reported a very high amount (57–168 h/week). Most dyads were in couple (51.9%) or adult–child relationships (40.3%) and most dyads lived together (54%). About 45% reported that other persons from their social environment were involved in the provision of care and support. The distribution for the most relevant of the class-forming indicators (R^2^ close to 50% and above) are comparable for informal caregiver’s age, the age of individuals with dementia, informal care relationship, sex of individuals with dementia and paid work of informal caregivers. The latter if full-time and part-time work are combined in the original study. Relevant differences exist between the indicators sex of informal caregivers (33.4% male caregivers in this study compared to 25% in the original study) and living situation (61.2% living together in this study compared to 54% in the original study).

### Fit statistics

Models with different numbers of classes (1–9) were compared and, consistent with the original study, a 6-class solution fitted the data best (for comparison see supplementary table 4 and supplementary table 5, additional file 2). In both LCA, the decision for the best model was based on fit indicators (BIC, LRT, Entropy) and on meaningful interpretability. Like the original study, the replication study shows a 3-class model with good fit statistics as well, especially regarding model improvement. Strong statistical improvement can be observed comparing the 2-class and the 3-class solution and only slight improvements in comparison of the models with 4, 5 or 6 classes (see Table 3). In the case of the 3-class solution, one could be inclined to think that adding another class doesn't give much better modeling of the data. Nevertheless, fit statistics prefer the 6-class solution over the 3-class solution. Furthermore, the 3-class model might be a too simple typology, as it cannot reveal significant differences between the dementia care dyads. Neither regarding the age of individuals living with dementia and informal caregivers nor the importance of the occupational situation of the informal caregivers.

**Table 3:** Replication LCA: Model fit evaluation information for k-class model

	**1-Class**	**2-Class**	**3-Class**	**4-Class**	**5-Class**	**6-Class**	**7-Class**
Log-Lik (LL)	-3103,14	-2594,37	-2425,64	-2394,04	-2359,69	-2320,66	-2304,10
BIC (LL)	6293,53	5325,86	5038,26	5024,91	5006,07	4977,87	4994,61
Entropy Score	1.00	0.9848	0.9878	0.9414	0.8710	0.8716	0.8741
LRT	-	< 0.001	< 0.001	< 0.001	< 0.001	< 0.001	< 0.001

### Indicator variables

In total, 17 indicators were included. Of these 17, twelve indicators contributed significantly (*p* < 0.05) to the best-fitting 6-class model. Table 4 shows all indicator variables included in the replication analysis. Compared to the original LCA, fewer indicators contributed significantly to the final model solution with six classes. Whereas in the original study 15 out of 16 indicators were significant, in this study there were only twelve out of 17 indicators significant with the variables *start of dementia symptoms*, *ADL (Katz-15)*, *NPI agitated*, *MMSE* and *RSES* not significant (n.s., gray background in table 9). We used the indicators whose variance was sufficiently explained by the best-fitting model (R^2^ ≥ 0.3, bold in Table 4) to match the approach of the original LCA.

**Table 4:** Indicator variables used for replication LCA with Wald, p-value and R^2^

Indicator variable	Wald	*p*-value	R^2^
Age individuals living with dementia	104,6434	5,50E-21	**0.526**
Age informal caregivers	90,087	6,40E-18	**0.811**
Informal care relationship	46,8625	1,00E-06	**0.812**
Sex individuals living with dementia	50,6266	1,00E-09	**0.665**
Sex informal caregivers	26,5948	6,80E-05	**0.535**
Living situation	173,0228	1,70E-35	**0.758**
Further informal support	71,4631	5,10E-14	0.160
Paid work informal caregivers	123,2068	6,60E-25	**0.490**
Education individuals living with dementia	34,0844	2,30E-06	0.079
Time informal caregiver spend for care and support	51,7255	6,10E-10	0.145
NPI aggression	14,5619	0,012	0.031
NPI inappropriate	20,6745	0,00,093	0.053
Start of dementia symptoms	7,4936	0,19 (n.s.)	0.021
ADL (Katz-15)	5,675	0,34 (n.s.)	0.020
NPI agitated	5,0602	0,41 (n.s.)	0.010
MMSE	2,9842	0,7 (n.s.)	0.008
Regional Socio-economic status (RSES)	4,6637	0,46 (n.s.)	0.013

A crucial step in evaluating the results of a LCA is to label and describe the statistically determined different classes in a concise heuristic way using key indicators of the LC model. For this, the most important class-forming indicators were used. Classification is based on the most likely class membership for each case.

### Dementia care dyad classes identified

**Fig. 1:**
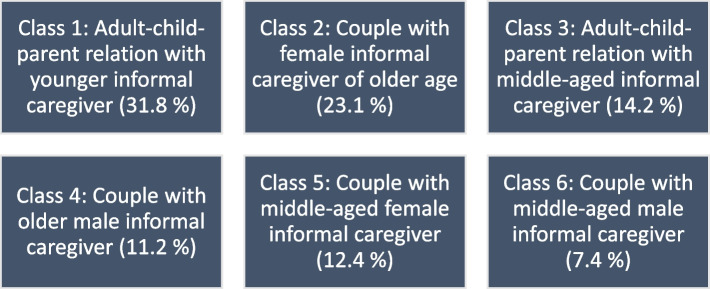
Six dyad classes identified via replication LCA (class size in parentheses)

As in the German sample, the best fitting model of the Dutch sample included six informal dementia care dyad classes (see Fig. 1). Some classes’ key characteristics which also emerged in the original study appear again in this replication study. However, some features could not be replicated due to sample specifics and difficulties in harmonizing the data used. The classes and class building characteristics of both studies can also be taken from supplementary table 6 and table 7 (Additional file 2).

Class 1 (class size: 31.8%), labelled as “adult–child-parent relationship with younger informal caregiver” is characterized by intergenerational child-parent relationships. In both studies, we find the youngest group of informal caregivers in this class (mean: approx. 51 years in both studies). The individuals with dementia of this class have a mean age of 81.1 years. Other similarities are that dyads typically do not live together, and that the informal caregivers have a paid job. Nevertheless, there are also differences. For instance, in the Dutch sample this group shows a larger proportion of male informal caregivers, although female informal caregivers are still dominant. Furthermore, this class is the most prevalent in the Dutch sample, in contrast to the German study (22.9%) where it is the second-largest class.

Class 2 (class size: 23.1%) is composed of older female informal caregivers (mean: 75.9 years) being in an intragenerational couple relationship with male individuals with dementia (mean: 80.5 years). Individuals are slightly older than in the original LCA (individuals with dementia and informal caregiver, approx. 2 years). The dyads live together, and informal caregivers are characteristically retired. This pattern is very close to that of Class 5 in the original study, so it was labelled “couple with female informal caregiver of older age” accordingly. As for the prevalence, this type is slightly less prominent in the current study (largest class with 31.4%) compared to the original study.

Class 3 (class size: 14.2%) has less clear overlap with any class from the original study. In a comparative general assessment of the six classes, the informal caregivers of this type are middle-aged (mean: 62.2 years) and the individuals with dementia are the oldest (mean: 88.7 years). The age difference indicates the intergenerational relationship constellation of informal caregivers as adult–child and individuals with dementia as parents, both predominantly female. The age and relationship structure plus the tendencies in the gender constellation point to similarities with Class 3 of the original study, accordingly we titled this class “adult–child-parent relationship with middle-aged informal caregiver”. The classes have a quite similar size. In the Dutch sample, this class includes more men as informal caregiver (28.5%), probably due to the overall larger proportion of male informal care (33.4%). Furthermore, fewer dyads are living together in this class (7%) compared to Class 3 in the German sample.

Class 4 (class size: 11.2%) is characterized by dyads in couple relationships with male informal caregivers and female individuals living with dementia. Informal caregivers (80.5 years) and individuals with dementia (80.1 years) are both at a very old age. Since this class is similar to class 4 of the original LCA, we labelled it “couple with male informal caregiver of older age”. The dominant form of housing is that of the shared household, and the majority of informal caregivers are retired (98%).

Class 5 (class size: 12.4%) also consists of dementia care dyads in couple relationships. The prominent differences between this class and class 4 are that informal caregivers are of female sex and both, individuals with dementia (-10.9 years) and informal caregivers (-17.6 years) are far younger. Because of these differences, we named this dyad type “couple with middle-aged female informal caregiver”. As in class 2 and class 4, the dyads are living together. A similar group with these characteristics and the dyadic age structure (slightly less than one third of the informal caregivers have a paid work) was not detected in the original LCA.

Class 6 (class size: 7.4%) is the cluster with the youngest individuals with dementia (68.2 years). The dyads are living together in couple relationships, and it is the second class in the Dutch sample with male informal caregivers. This class was labelled as “couple with middle-aged informal caregiver”. Even though it is a class with quite young individuals living with dementia, it’s not a class of young couples like class 6 from the original study. As in class 5 of the current LCA, there is a similarly large proportion of working informal caregivers.

### Correlation of classes with health care outcomes (distal outcomes)

After the identification of the best fitting LCA model, the associations between the six classes and several outcome measures were examined.

#### Use of information sources

Regarding the use of medical services (i.e., general practitioner, hospital) as sources for getting information, there are significant differences between the six classes (*p* = 0.0064). Similarly, there are significant differences (*p* = 0.001) for the use of civil society sources (i.e., informal care organization, Alzheimer Netherlands). There is no significant difference (*p* = 0.14) in terms of getting information via nursing services (i.e., day-care meeting center, community advisor for older individuals) (see Table 5).

**Table 5:** Association of classes and use of information sources and health care services bases on most likely class membership

Distal outcomes	Classes						*p*-value (Wald-Test)
	**1**	**2**	**3**	**4**	**5**	**6**	
Class size (%)	*31.8*	*23.1*	*14.2*	*11.2*	*12.4*	*7.4*	
Information source: Medical	60.5%	58.2%	34.0%	71.0%	54.5%	79.0%	**0.0064**
Information source: Nursing	50.1%	43.6%	47.7%	31.8%	48.1%	73.6%	0.14 (n.s.)
Information source: Civil Society	31.3%	30.3%	24.8%	34.5%	64.5%	58.1%	**0.001**
Health care service: Medical	94.6%	85.3%	90.3%	89.7%	93.4%	92.8%	0.46 (n.s.)
Health care service: Therapeutic	27.1%	34.9%	25.4%	26.5%	34.2%	39.8%	0.66 (n.s.)
Health care service: Nursing	82.4%	64.7%	96.0%	58.6%	54.1%	56.4%	** < 0.001**

#### Health care service use

The use of medical health services (*p* = 0.46) as well as the use of therapeutic services (*p* = 0.66) shows both no significant differences between the six dyad classes. Regarding nursing services, the classes differ significantly (*p* < 0.001).

#### Quality of life (Qol-AD)

The Qol-AD differs significantly between the classes (*p* < 0.001). The informal caregivers in couple relationships (classes 2, 4, 5, 6) rate the QoL of their relatives with dementia consistently better than the informal caregivers in adult–child-parent relationships (classes 1, 3). The worst QoL scores for individuals with dementia are reported by informal caregivers of class 3 (adult–child-parent relation with middle-aged informal caregiver) and the best QoL scores for individuals with dementia of class 6 (couple relation with middle-aged male informal caregiver).

#### Health status of informal caregiver (GHQ-12)

There are significant differences between the dyad classes regarding the health status of the informal caregivers (*p* < 0.001). The worst health scores are reported for classes 2 and 5, both groups saliently characterized by female informal caregivers living together with male individuals with dementia in couple relationships. In class 3, which is, despite its more mixed character, a type with predominantly female informal caregivers in adult–child-parent relationships, the best GHQ-12 scores are reported.

#### Caregiver burden (CarerQoL)

To examine burden in a differentiated way, the CarerQoL subscales were used. The result is mixed with domains differing significantly between the classes as well as non-significant domains. On the one hand, the domains focusing on problems with individuals living with dementia (i.e., communication) (*p* = 0.048)*,* problems with own physical health (*p* = 0.002), receiving support from family/friends etc. (*p* < 0.001) and problems with own mental health (*p* < 0.001) show significant inter-class differences. On the other hand, there are no significant differences regarding domains such as satisfaction performing care duties (*p* = 0.14), difficulties to combine daily activities (*p* = 0.22) and financial issues with care duties (*p* = 0.33). Informal caregivers of class 2 (older female informal caregivers in couple dyads) report the most severe burden of physical and mental health problems.

#### Number of unmet needs (CANE)

The number of unmet needs differed significantly between the six classes (*p* = 0.031). Most unmet needs are recorded for the classes 1 (mean: 1.9) and 3 (mean: 1.8), both dyad types in intergenerational adult–child-parent relationships, not living together and with relatively young informal caregivers, especially in class 1. The fewest unmet needs (mean: 1.1) are reported in class 2, with female informal caregivers of older age in couple relationships. Table 6 summarizes results regarding Qol-AD, CarerQoL, GHQ-12 and CANE unmet needs. Since subscales and not the total score of the CarerQoL are of interest, CarerQoL subscales are listed in the table.

**Table 6:** Association of classes and Qol-AD, CarerQoL, GHQ-12 and CANE based on most likely class membership

Distal outcomes	Classes						*p*-value (Wald-Test)
	**1**	**2**	**3**	**4**	**5**	**6**	
Class size (%)	*31.8*	*23.1*	*14.2*	*11.2*	*12.4*	*7.4*	
QoL-AD (3–52; higher score = better QoL)	29.5	31.2	28.3	32	31.1	32.6	** < 0.001**
CarerQoL (in %, reference category “yes”)							
* Satisfaction performing care duties*	86.6	91.4	94.6	94.0	85.4	87.5	0.14 (n.s.)
* Problems with person with dementia*	54.5	72.8	57.7	64.0	68.2	73.8	**0.048**
* Problems with own mental health*	36.5	67.5	30.9	30.9	62.5	53.8	** < 0.001**
* Problems with own physical health*	35.8	59.8	35.7	41.3	58.0	38.7	**0.002**
* Problems to combine daily activities*	49.1	55.9	46.4	30.8	53.3	48.6	0.22 (n.s.)
* Financial problems with care duties*	7.6	15.5	5.6	1.9	21.8	29.1	0.33 (n.s.)
* Receiving support from family/friends *etc	85.8	71.5	84.0	78.3	57.3	68.7	** < 0.001**
GHQ-12 (0–36; higher score = worse health)	11.8	14.7	10.4	11.9	14.7	13.3	** < 0.001**
CANE (0–26; higher score = more unmet needs)	1.9	1.1	1.8	1.3	1.2	1.6	**0.031**

### Original LCA vs. replication LCA: Comparison of class model and class characteristics

#### Class enumeration

In both studies, the decision on the best model was based on the statistical fit indices BIC, Entropy and LRT. Furthermore, the best statistical solution also needed a robust interpretation. Following this harmonized approach, both studies yielded a 6-class model solution that fitted the data best.

#### Class characteristics

Concerning the characteristics that formed the six classes, this study shows similarities but also differences between the German and the Dutch sample (see supplementary table 6 and supplementary table 7, additional file 2). Overall, there were three couple and three adult–child-parent classes in the original study, whereas in the current study four couple classes and two adult–child classes were identified.

Although there are some minor differences with regard to single characteristics (i.e., more male informal caregivers in the Dutch sample), class 1 of the Dutch sample is very similar to class 1 of the German sample. Structurally analogous in terms of content, they differ in terms of their prevalence. This type occurs more frequently in the Dutch data set (+ 8.9%-points). Class 2 of the current study resembles class 5 of the original study. Structurally similar, they slightly differ regarding the age of informal caregivers and individuals living with dementia, with both individuals a little older in the Dutch sample. Even though there are differences in terms of sex ratio (more male individuals) and the proportion of dyads living together, class 3 of the Dutch sample and class 2 of the German sample are comparable. Different from the original German sample, with only one distinct cluster with male informal caregivers (class 4), this LCA identified two male informal caregiver classes (classes 4 and 6). Those two Dutch male classes differ considerably with respect to the mean age of individuals with dementia and informal caregivers and, derived from this, also regarding the proportion of working informal caregivers (higher percentage in class 6). The fact that two separate clusters with male caregivers emerge in this replication study may be related to the overall higher proportion of male informal caregiver and its increased age-related diversity in the Dutch sample. Class 5 of the current study has no similarities to one of the classes from the original study. The original LCA showed a distinct couple class, with female informal caregivers as the most prevalent class (31.4%). In the Dutch sample, a differentiation within the group of female informal caregivers in couple relationships can be observed, which results in two separate types (classes 2 and 5). Similar to the differentiation of the couple classes with male informal caregivers, the two couple classes with female caregivers differ considerably regarding the mean age of both, the individuals with dementia and the informal caregivers.

In the original German LCA two classes could be identified that do not emerge in the Dutch LCA. First, a small class (class 3, size: 8.8%) with predominantly non-kinship and distance caregiving relation and second, a class with younger couples (class 6, size: 5.8%).

### Original LCA vs. present LCA: Comparison of distal outcomes

In the following sections, the distal outcomes (health care related outcomes) of both LCAs are presented comparatively. For a better overview, we included all relevant tables in the additional material, even if they are already included in the text (see supplementary tables 8–11, additional file 2).

#### Use of information sources and health care services

In terms of using medical services for gathering information, there are significant differences between the classes in both samples. Another common feature is that the use of nursing services is non-significant neither in the original nor in the replication LCA. A contrasting result can be noted with regard to the civil society sources: In the current study there are significant differences between the six groups, but not in the original study. The use of medical and therapeutic health care services shows no differences between the classes in the current study, but both service types differed significantly in the original study. Regarding nursing services, there are significant differences in both studies.

On a more general level, like in the original study, the use of nursing health care service is considerably more prevalent in dyads with intergenerational adult–child-parent relationships.

#### Quality of Life of individuals living with dementia

For both samples, the differences between the dyad classes regarding quality of life (Qol-AD as proxy measure in both studies) are significant. The overall mean in the Dutch sample is 1.6 points higher (means better QoL) than in the German sample (28.7 vs. 30.3). As in the original study, the quality-of-life scores are better in couple relationships than in adult–child-parent relationships. In both, the original study and this replication study, the lowest QoL scores were reported by adult–child informal caregivers in the classes with the oldest group of individuals living with dementia.

#### Health status of informal caregivers

The health status scores for informal caregivers, measured via EQ 5D VAS in the original study and via GHQ-12 in the replication study, show few similarities in both studies. A common characteristic of both 6-class models is, that the worst health status of the informal caregivers occurred in the structurally similar classes 2 (Dutch sample) and 5 (German sample). In both studies, this class is among the largest two and thus especially relevant. Furthermore, in the original study as well as the replication study, health scores are worst in dementia dyads with couple relationships. For the original study this applies to all couple relationship classes, in the current study for the couple classes with female informal caregivers.

#### Caregiver burden

In the current study, four out of seven CarerQoL subdomains show significant differences between the classes. Measured via BIZA-D subscales in the original study, caregiver burden differed in all subdomains. In both studies, classes with higher mean caregiver burden scores also show worse caregiver health scores.

#### Stability of care arrangement vs. unmet needs

The CANE unmet needs scale was used as a proxy for determining the stability of the home-based care arrangement. Using these two distinct instruments as comparative tools, makes it difficult to analyze cross-study patterns. Nevertheless, it might be noteworthy that the results of the structurally similar class 1 (“adult–child-parent relationship with younger informal caregiver”) of both samples point in the same direction: In the original study, this class is the "least stable" class, and in this replication study it is the class with the most unmet needs. This parallel can also be observed for the similar classes 2 of the original study and class 3 of the current study (“adult–child-parent relation with middle-aged informal caregiver”) with some limitations.

## Discussion

The aim of this study was to examine whether the subgroup structure and association with healthcare outcomes found in the original German study could be replicated in a different Dutch sample. For this purpose, the Dutch data of the present study was prepared to match the data from the original German study and the same statistical approach was applied. From a methodical perspective, a key challenge for replication of studies, is the heterogeneity of the datasets being compared. Even though some general patterns emerge, LCA still is a data-driven approach, where non-conformity of indicator variables used influences the class structures that can be identified [58]. This is also the case for the present study, even if data were matched as close as possible to the original LCA. Not all the indicators of the original LCA study could be filled with identical indicators for this replication study. It is recognized, however, that complete replication is not possible, as every replication assesses the generalizability to the specific context of the new study [32]. However, the findings of the present study confirm those of the original LCA and emphasize that relevant subgroups of informal dementia care dyads exist.

### Class enumeration and class characteristics

Both studies yielded in a best-fitting 6-class model. This overall result is not surprising given the structural similarity of the data sets. Both the size of the data sets and the basic similarity of the main class-forming indicators can be seen as the reason why the statistical metrics (BIC, LRT, Entropy) as well as the interpretability, led to the decision for a 6-class model in both studies. Although the number of classes is the same, substantive differences still exist concerning class sizes, class-forming indicators, and associations with health-related outcomes due to the heterogeneity of the two data sets and populations.

As in the original study, the indicator variables informal care relationship (R^2^ = 0.812) and age of informal caregivers (R^2^ = 0.811) are those with the highest explained variance in the LC model of the current study. Analogous to the German LCA, we have thus used them for the labelling of the six Dutch classes. There is nothing novel in pointing out that couple and adult child-parent relations are the dominant forms of informal dementia care dyad relationships. This is already confirmed by earlier studies [59]. While three couple and three adult–child-parent subgroups were identified in the original study, there are four couple groups and two adult–child-parent groups in the present study. Having one more group of couples identified may be because more male caregivers participated in the Dutch replication study than in the original German study and that they are more age diverse, so that two male caregiver classes emerged in the present study.

The most prevalent class of the original LCA was a couple class with female caregivers of older age (31.4%) and an adult–child caregiver class with younger informal caregivers in the present study (31.8%). The original German LCA gave the indication of a small class (class 3, size: 8.8%) with a predominantly non-kinship and distance caregiving relation. Besides, a class with younger couples indicating early onset dementia situations was part of the original study (class 6, size: 5.8%). Due to methodological and population reasons, both classes could not be replicated within the present study.

### Health care related outcomes

Regarding health care service use, in both studies, dyads with intergenerational adult–child-parent relationships use more frequently nursing health care services. This is less surprising, as in these dyad classes, the individuals living with dementia are the oldest. Studies indicate that it is more likely that dementia is more advanced in older age groups (over 80 years) leading to increased health problems and more professional nursing assistance being sought [60]. In addition, adult–child informal caregivers rarely live together with the individuals with dementia, so they might be less likely to take on continuous and extensive caregiving responsibilities and are more likely to have professional help involved in the care arrangement [61].

A common pattern of both studies can be seen regarding the general health status of the informal caregivers. Health scores are worse in dyads with couple relationship and worst in the structurally similar classes 2 (Dutch sample) and 5 (German sample), both classes in couple relations with the oldest female caregiver. Other studies also highlight these patterns and conclude that dyads with couple relationships are characterized by a higher degree of closeness. Adult–child caregivers typically do not live in the same household and are less intensively involved in caregiving, which might result in less negatively affected health status. In addition, it is not uncommon for informal caregivers in dyads with couple relationships to be at an older age themselves, which means that the probability of own health problems is increased [59, 62]. However, the relationship between increasing age and worsening health status is not a dementia-specific link.

The worst health status scores for informal caregivers are accompanied by the highest caregiver burden scores in both studies. This relates to informal caregivers in couple relationships, particularly for older female caregiver. The combination of these two adverse health markers indicates groups with particular risk profiles that need to be specifically addressed by support services, an issue that has already been reported [63].

Even though instruments used differ conceptually, it is interesting that the class with the most unstable care arrangement in the German sample, is at the same time the class with the most unmet needs in the Dutch sample. In both studies, this is the group with working caregivers, indicating the need for targeted support that considers the specific challenge of informal caregivers to reconcile work and care. When designing care plans, it is important to consider both restrictions and resources resulting from the occupation situation of the informal caregivers [64]. Possibilities of integrating employers into the design of care plans aimed at reconciling care and work should also be considered. For example, by promoting programs to make working time models more flexible or in-house courses to promote caregiver health [65]. Since it is not uncommon for female caregivers to reduce their work time or give up paid work due to the high amount of care they provide, it is essential to keep an eye on the negative effects on income and retirement income in the context of counseling strategies [66, 67]. In both, the original study and this replication study, the lowest QoL scores are reported by adult–child informal caregivers in classes with the oldest individuals with dementia and better QoL scores in dyads with couple relationships that live together. Since QoL is a key outcome in the care of individuals with dementia, particular attention must be paid to groups with relatively low QoL [68, 69]. The results discussed indicate, that counseling may play a pivotal role in the identification of dyad subgroups and in the provision of tailored health care services. Studies point out, that counseling embedded within a community-based case and care management program can help to provide efficient, needs-based services to people with dementia and their informal caregivers [70].

### Strengths and limitations

The strengths of the present study are, among others, the transparent methodical and statistical approach and the detailed reporting. This can serve as a basis for critical appraisal and further replication. Moreover, with the effort to replicate a LCA with a different sample to prove if the results of the first study are reliable, we have faced the criticism of LCA that is a somehow subjective approach. However, this study also has limitations. As noted above, the present replication of the original DemNet-D LCA using the COMPAS data was partially limited. This is mainly due to variations in study design and the way data has been collected in both studies (i.e., living situation, informal care relationship, paid work/occupation). In addition, variables (regarding regional care structures) that could be included in the German LCA were not available in the Netherlands. There are certainly issues regarding the generalizability of the study results because both data sets are taken from populations that were part of dementia care net structures. More general conclusions might be drawn if individuals living with dementia and informal caregivers with more diverse access to health and care structures were included. It would also be important to replicate latent class structures with data from different countries and world regions with different health and care systems to verify the general validity. As in the original LCA, the present replication study used cross-sectional data, and it would be interesting for future research to focus on longitudinal analyses, i.e., how clinical outcomes within different dyad subgroups change over time.

## Conclusion

This study demonstrates, in two structurally similar but different data sets, that relevant subgroups of dyadic dementia care constellation exist. In both samples, these can be meaningfully distinguished based on a core set of indicator variables (age person with dementia, age informal caregiver, sex person with dementia, sex informal caregiver, informal care relationship, living situation, occupation informal caregiver). In both LCA studies, differences between dyad subgroups regarding relevant healthcare outcomes (i.e., quality of life of individuals with dementia and health status, burden and unmet needs of informal caregivers) implies that tailoring support services to certain life and care situations of dementia care dyads is of key importance. The observed differences provide useful contributions for a more tailored design of health promotion services for informal caregivers and individuals with dementia in community-dwelling settings, and underline the relevance of a subgroup specific dyadic perspective. In most previous intervention studies, informal dementia care dyads appear as a fairly homogeneous group sharing similar care contexts and needs. However, the results of the present study suggest that these assumptions should be carefully reviewed regarding an effective design and implementation of counseling and support services. To have a significant impact on improving care situations of individuals with dementia and informal caregivers, future health care and support structures will require tailoring to specific living and care situations. These tailored services should be implemented as innovative elements of existing local or regional care systems. Furthermore, broadening the dyadic perspective to include the wider social network would be an important departure for further research. For future research, it would be conducive to use a basic set of sociodemographic indicators that is generally accepted, so that individual findings can be replicated more accurately.

## Supplementary Information

**Additional file 1.**

**Additional file 2.**

## Data Availability

The data that support the findings of this study are available from the corresponding author, HW, upon reasonable request.

## Abbreviations

LCA: Latent Class Analysis
LC: Latent Class
QoL: Quality of Life
MCI: Mild Cognitive Impairment
U.S.: United States
QoL-AD: Quality of Life in Alzheimer's Disease
SACA: Sense of Acceptance in Community Activities
BIZA-D: Berlin Inventory of Caregivers' Burden with Dementia Patients
EQ-VAS: EuroQoL Group Visual Analogue Scale
SoCA: Stability of Care Arrangements
OECD: Organization for Economic Co-operation and Development
DemNet-D: Evaluation study of dementia networks in Germany
STROBE: Strengthening the Reporting of Observational Studies in Epidemiology
DCN: Dementia Care Networks
ADL: Activities of Daily Living
NPI: Neuropsychiatric Inventory
CMAI: Cohen-Mansfield Agitation Inventory
MMSE: Mini Mental Status Examination
RSES: Regional Socio-Economic Status
GHQ-12: 12-Item Short-Form Health Survey
CarerQoL: Carer Quality of Life
CANE: Camberwell Assessment of Need for the Elderly
FFM: Finite Mixture Models
BIC: Bayesian information criterion
LRT: Likelihood Ratio Test
EM: Expectation–Maximization
ML: Maximum likelihood
MAR: Missing at random
SD: Standard Deviation
n.s.: Not significant
