# Supplementary material for: Sociodemographic structure and health care-related outcomes of community-dwelling dementia caregiving dyads: a latent class replication study
Source: BMC Health Serv Res. 2023 May 12;23:482. doi: 10.1186/s12913-023-09505-5 (PMC10182645; doi:10.1186/s12913-023-09505-5)
Supplement: Supplementary file 2 — Additional file 2. [file 12913_2023_9505_MOESM2_ESM.docx]

Supplementary table 1 Comparison of indicator variables used in original LCA vs replication LCA

| Domains | Measures original LCA (DemNet-D data; n=551) | Coding | Measures replication LCA  (COMPAS data; n=509) | Coding |
| --- | --- | --- | --- | --- |
| Individuals living with dementia | | | | |
| Age | Age grouped | 1=under 70 years  2=70-74 years  3=75-79 years  4=80-84 years  5=85-89 years  6=90 years and older | Age grouped | 1=under 70 years  2=70-74 years  3=75-79 years  4=80-84 years  5=85-89 years  6=90 years and older |
| Sex | Sex, binary coded | 0=male  1=female | Sex, binary coded | 0=male  1=female |
| Functional independence | Instrumental activities of daily living (IADL, Lawton & Brody Scale)  (proxy, informal caregiver) | 1=lower function, more dependent (0-2)  2=higher function, less dependent (3-8) | Activities of daily living (Modified KATZ-15 ADL)  (proxy, informal caregiver) | 1= lower function, more dependent (5-15)  2= higher function, less dependent (0-4) |
| Behavioural symptoms | Cohen-Mansfield Agitation Inventory (CMAI)  Single items used: aggression, agitated, inappropriate  (proxy, informal caregiver) | Aggression: 0=not present, 1=present  Agitated: 0=not present, 1=present  Inappropriate: 0=not present, 1=present | Neuropsychiatric Inventory (NPI)  Single items used: aggression, agitated, inappropriate  (proxy, informal caregiver) | Aggression: 0=not present, 1=present  Agitated: 0=not present, 1=present  Inappropriate: 0=not present, 1=present |
| Dementia severity/Severity of cognitive impairment | Functional Assessment Staging Test (FAST),  (proxy, informal caregiver) | 0= no to mild cognitive impairment (1-4)  1=moderate to severe cognitive impairment (5-7) | Mini-Mental Status Examination (MMSE)  (self-reported, person with dementia) | 0=no to mild cognitive impairment (30-21)  1=moderate to severe cognitive impairment (20-0) |
| Informal caregivers | | | | |
| Age | Age grouped | 1=under 50 years  2=50-59 years  3=60-69 years  4=70-79 years  5=80 years and older | Age grouped | 1=under 50 years  2=50-59 years  3=60-69 years  4=70-79 years  5=80 years and older |
| Sex | Sex, binary coded | 0=male  1=female | Sex, binary coded | 0=male  1=female |
| Occupation | Q: Are you, in addition to the support and care of the person with dementia, employed? | 1=none  2=part-time  3=full-time | Do you have paid work? | 1=no  2=yes |
| Time spent on care and support | Q1: In the past 30 days, how much time on a normal day did you spend helping the person with dementia with care activities?  Q2: In the past 30 days, how much time on a normal day did you spend helping the person with dementia with activities such as shopping, preparing meals, housework, laundry, using transportation, taking medications, and taking care of financial matters?  Values Q1 and Q2 summed; both Q taken from Resource Utilization in Dementia instrument; RUD | 1=lower effort (≤2h)  2=moderate to high effort (2-8h)  3=very high effort (≥ 8h) | Q1: In the past week, have you had to help with household tasks due to his/her health problems?  Q2: Have you had to help with personal care during the past week due to his/her health problems?  Q3: In the past week, have you had to help because of his/her health problems when moving outside, contacts with health care, administration?  Values Q1-3 summed; Three Q single items from COMPAS questionnaires | 1=low effort (0-14 h/week)  2=moderate to high effort (15-56 h/week)  3=very high effort (57-168 h/week)  Staging in COMPAS LCA adapted to staging in original study, which was extrapolated from daily to weekly values. |
| Duration of care | Since when are you involved in the care of the person with dementia? | 1= short to medium duration of care (≤1 years)  2=long duration of care (≥4 years) | How long ago did symptoms of dementia start (months)? | 1= up to 50 months  2= more than 50 months |
| Dyad level | | | | |
| Informal care relationship | What is your relationship to the person with dementia you care for? | 1=couple,  2=adult-child  3=other/non-kinship | What is your relationship with the person living with dementia? | 1=couple  2=adult-child  3=other/non-kinship |
| Living situation | Do you live together? | 1=living together  2=not together but close  3=other/further away | Do you live together? | 1= yes  2= no |
| Socio-economic status (SES) | Scheuch-Winkler Index  (Composite SES measure based on income, education, (former) occupation of person with dementia) | 1=lower social class  2=middle social class  3=high social class | Education of person with dementia as proxy for SES  Original staging:  1=less than 6 classes primary school  2=six classes primary/lom-school/mlk-school  3=more than 6 classes primary school, FU not completed  4=middle level education  5=higher secondary education (Hbs/gymnasium/atheneum)  6=university/higher education  Recoded to 1-3 staging to adapt it to PFADE LCA | 1= low education (1,2)  2= medium education (3,4)  3= high education (5,6) |
| Informal support | Q: Are other persons (family, friends, neighbors) involved providing care? | 0=no  1= yes | Q: In addition to you, does (name) also receive help from other caregivers or volunteers? | 0=no  1=yes |
| Regional level | | | | |
| Regional socio-economic differences | German Index of Socio-economic Deprivation (GISD) | 1=low level of socioeconomic deprivation  2=medium level of socioeconomic deprivation  3= high level of socioeconomic deprivation | Regional socio-economic status (RSES) (postal code)  Socio-economic status scores are calculated by The Netherlands Institute of Social Research (Sociaal Cultureel Planbureau - SCP) and indicate the relative social status of a postcode-4 district compared to other postcode-4 districts in the Netherlands and are based on population averages regarding education, income and position in the labor market. See Knol (2012) and SCP (2019) for more details. | 1=lower third  2=middle third  3=upper third  Staging based on distribution to three equal ranges:  <= -,22 (lower third)  -,21 - ,63 (middle third)  ,64+ (upper third) |
| Regional care infrastructure | Regional outpatient care quota | ≤8.2: below-average care quota  >8.2: above average care quota^^[[1]](#footnote-1)^^ | No comparable data. | - |
| Medical and therapeutic infrastructure | Regional physician quota | ≤63.9: below-average physician quota  >63.9: above-average physician quota^^[[2]](#footnote-2)^^ | No comparable data. | - |

Supplementary table 2 Comparison of distal outcomes used in original LCA vs. replication LCA

| Domains | Distal outcomes used  in original LCA  (DemNet-D data; n=551) | Coding | Distal outcomes used in replication LCA  (COMPAS data; n=509) | Coding |
| --- | --- | --- | --- | --- |
| Informal caregivers | | | | |
| Care burden | BIZA-D Subscales; Burden due to…   1. cognitive losses 2. aggression/resistance 3. personal constraints/health 4. lack of social support | 0-16  0-20  0-20  0-24 | CarerQoL (self-rated):   1. Problems physical health 2. Receiving informal support 3. Financial problems 4. Problems combining daily activities 5. Problems mental health 6. Relational problems with person with dementia 7. Satisfaction performing care duties | Items a-g staged as follows:  0=no  1=some  2=a lot |
| General Health | General health status (EQ-VAS)  (self-rated) | 0-100 | General Health Questionnaire (GHQ-12) (self-rated) | 0-36 |
| Individual living with Dementia | | | | |
| Quality of Life | Quality of Life-Alzheimer’s Disease (QoL-AD)  (proxy, informal caregiver) | 13-52 | Quality of Life-Alzheimer’s Disease (QoL-AD)  (proxy, informal caregiver) | 13-52 |
| Social participation | Social Acceptance in Community Activities (SACA) | 1-32 | No similar instrument in COMPAS data set | - |
| Dyad level | | | | |
| Health care  service use I | Health care service use: Information sources   1. Informal sources 2. Medical sources 3. Nursing sources 4. Civil society sources | no use, use  no use, use  no use, use  no use, use | Q: How often in the last 3 months did you use info from (self-rated, informal caregiver)   1. Medical sources: General practitioner, Hospital, Pharmacy 2. Nursing sources: Community advisor for older persons, Daycare-Meeting-Centre, Office for care assignment, CIZ 3. Civil society sources: Alzheimer Netherlands, Organization for informal care | 1=yes, used  0=no, not used |
| Health care  service use II | Health care service use: Professional care   1. Medical services 2. Therapeutic services 3. Nursing services 4. Support services | no use, use  no use, use  no use, use  no use, use | 1. Medical services: Has your loved one had contact with the general practitioner or practice nurse? Did your closest person receive a visit at home from a specialist geriatrics doctor (nursing home doctor)? 2. Therapeutic services: Has your loved one been in contact with a therapist? 3. Nursing services: Day care - Did your closest one have day care or used day care in another location? Has your loved one had night care in an institution other than a hospital? Has your loved one received home care? | 1=yes, used  0=no, not used |
| Stability of the care situation | What is your overall assessment of the care situation this month?  (self-rated, informal caregiver) | 0=care at home does not work anymore (..),  1= urgent need for more help,  2=situation is well arranged, if dementia progresses, more help is needed  3= situation is well arranged, even if situation worsens | Total number of unmet needs (CANE needs assessment)  (self-rated, informal caregiver) | 0-26 |

Supplementary table 3 Sample description original German LCA vs. Dutch replication LCA

| **Original LCA**  **(DemNet-D data; n=551)** | | | **Replication LCA**  **(COMPAS data; n=509)** | | | |
| --- | --- | --- | --- | --- | --- | --- |
| **Individual living with dementia** | | | **Individuals living with dementia** | | | |
| Age | in years | 79.5 (8.5) | Age | in years | 79.7 (7.9) |  |
| Sex | female (ref.) | 57.0 % (316) | Sex | female (ref.) | 55.0 % (280) |  |
| Social class | low  middle  high  no information | 44.4 % (245)  21.8 % (120)  7.1 % (39)  26.7 % (147) | Education | low  middle  high  no information | 21.8 % (111)  54.6 % (278)  18.3 % (93)  5.3 % (27) |  |
| Dementia severity (FAST) | no-mild (1-4)  moderate-severe (5-7)  no information | 6.6 % (36)  90.2 % (497)  3.3 % (18) | Severity of cognitive impairment (MMSE) | no-mild (30-21)  moderate-severe (20-0)  no information | 42.2 % (215)  28.3 % (144)  29.5 % (150) |  |
| Instrumental Activities  of Daily Living (Lawton/Brody IADL) | more dependent (0-2)  less dependent (3-8)  no information | 62.7 % (345)  28.4 % (157)  8.9 % (49) | Activities of Daily Living (Katz ADL-15) | more dependent (5-15)  less dependent (0-4)  no information | 73.1 % (372)  22.6 % (115)  4.3 % (22) |  |
| Cohen Mansfield Agitation Inventory (CMAI)  inappropriate  aggressive  agitated | yes, present  yes, present  yes, present | 61.7 % (340)  15.2 % (84)  58.8 % (324) | Neuropsychiatric Inventory  (NPI)  inappropriate  aggressive  agitated | yes, present  yes, present  yes, present | 31.4 % (160)  40.1 % (204)  41.7 % (212) |  |
| QoL-AD | 3-52 | 28.7 (5.5) | QoL-AD | 3-52 | 30.3 (5.8) |  |
| SACA | 1-32 | 24.4 (4.2) | - | - | - |  |
| **Informal caregivers** | | | **Informal caregivers** | | | |
| Age | in years | 64 (12.9) | Age | in years | 64.5 (12.5) |  |
| Sex | female (ref.) | 75.0 % (412) | Sex | female (ref.) | 66.6 % (339) |  |
| Occupation | full time  part time  none  no information | 14.3 % (79)  20.7 % (114)  62.4 % (344)  2.5 % (14) | Paid work | yes  no information | 38.9 % (79)  3.4 % (18) |  |
| Time spent for care | In hours per day | 4.7 (5.4) | Time spent for care & support (h/week) | 0-14 (low)  15-56 (moderate-high)  57-168 (very high)  no information | 48.9 % (249)  27.5 % (140)  6.9 % (35)  16.7 % (85) |  |
| Time spent for support | In hours per day | 4.6 (5.1) | - | - | - |  |
| Care duration | in years | 4.2 (3.6) | Start of dementia symptoms | up to 50 months ago  more than 50 months ago | 62.9 %  37.1 % |  |
| EQ VAS | 0-100 | 66.4 (19.6) | GHQ-12 | 0-36 | 13.0 (5.5) |  |
| BIZA-D Subscales:  Burden due to…  cognitive decline  aggression and resistance  personal restrictions  lack of social support | 0-16  0-20  0-20  0-24 | 8.1 (4.7)  6.0 (4.9)  8.3 (5.8)  7.7 (5.5) | CarerQoL:  Problems physical health  Receiving informal support  Financial problems  Problems combining daily activities  Problems mental health  Relational problems PwD  Satisfaction performing care duties | yes (some, a lot) (ref.)  yes (some, a lot)  yes (some, a lot)  yes (some, a lot)  yes (some, a lot)  yes (some, a lot)  yes (some, a lot) | 45.5 % (232)  73.9 % (376)  12.0 % (61)  48.7 % (248)  46.6 % (237)  61.5 % (313)  86,7 % (441) |  |
| **Dyad level** | | | **Dyad level** | | |  |
| Informal care relationship | couple  adult-child  other/non-kinship  no information | 50.6 % (279)  42.1 % (232)  6.7 % (37)  0.5 % (3) | Informal care  relationship | couple  adult-child  non-kinship/other  no information | 51.9 % (264)  40.3 % (205)  5.1 % (26)  2.8 % (14) |  |
| Living situation | living together  not together but close  other/further away  no information | 61.2 % (337)  22.3 % (123)  15.1 % (83)  1.5 % (89) | Living situation | living together (ref.)  no information | 54.0 % (275)  2.9 % (15) |  |
| Further informal support | yes (ref.)  no information | 88.0 % (485)  0.4 % (2) | Further informal support | yes (ref.)  No information | 45.0 % (229)  1.2 % (6) |  |
| Health care service use: Information sources  Informal  Medical  Nursing  Civil society | no, not used  no, not used  no, not used  no, not used | 28.9 % (159)  35.0 % (193)  44.8 % (247)  70 % (369) | Health care service use: Information sources  Medical  Nursing  Civil society | no, not used  no, not used  no, not used | 37.5 % (191)  45.4 % (231)  58.7 % (299) |  |
| Health care service use: Professional care  Medical services  Therapeutic services  Nursing services  Support services | no, not used  no, not used  no, not used  no, not used | 8.2 % (45)  69.7 % (384)  41.4 % (228)  68.2 % (376) | Health care service use: Professional care  Medical  Therapeutic  Nursing | no, not used  no, not used  no, not used | 7.3 % (37)  56.8 % (289)  22.4 % (114) |  |
| Stability of care arrangement | 0  1  2  3  no information | 3.3 % (18)  7.2 % (40)  51.9 % (286)  30.3 % (167)  7.3 % (34) | Number of unmet needs  (CANE) | 0-26 | 1.6 (2.1) |  |
| **Regional level** | | | **Regional level** | | | |
| Regional Socio-Economic  Deprivation (GISD) | low  middle  high | 7.2 % (40)  68.6 % (378)  24.1 % (133) | Regional Socio-Economic Status | low  middle  high | 33.4 % (170)  31.2 % (159)  31.8 % (162) |  |
| Regional care infrastructure (quota) | - | 7.8 (1.9) | **-** | **-** | **-** |  |
| Regional medical infrastructure (quota) | - | 63.5 (6.1) | **-** | **-** | **-** |  |
| Note: Categorical data is presented as (%)/N. Continuous data is presented as Mean/(SD). | | | | | |  |

Supplementary table 4 Replication LCA (COMPAS data): Model fit evaluation information for k-class model

| **Class** | **1** | **2** | **3** | **4** | **5** | **6** | **7** |
| --- | --- | --- | --- | --- | --- | --- | --- |
| Log-Lik (LL) | -3103,14 | -2594,37 | -2425,64 | -2394,04 | -2359,69 | -2320,66 | -2304,10 |
| BIC (LL) | 6293,53 | 5325,86 | 5038,26 | 5024,91 | 5006,07 | 4977,87 | 4994,61 |
| Entropy Score^*^ | 1.00 | 0.9848 | 0.9878 | 0.9414 | 0.8710 | 0.8716 | 0.8741 |
| LRT (bootstrap-p-value)^* *^ | - | <0.001 | <0.001 | <0.001 | <0.001 | <0.001 | <0.001 |
| *Entropy Score: This value indicates how distinctly the dyads can be assigned to the classes. The closer the value. is to 1, the better the model fit.  * * LRT: Likelihood ratio test for the model l with C classes vs. model with C-1 classes. | | | | | | | |

Supplementary table 5 Original LCA (DemNet-D data): Model fit evaluation information for k-class model

| **Class** | **1** | **2** | **3** | **4** | **5** | **6** | **7** |
| --- | --- | --- | --- | --- | --- | --- | --- |
| Log-Lik (LL) | -3460 | -2946 | -2773 | -2743 | -2711 | -2682 | -2672 |
| BIC(LL) | 7015 | 6031 | 5730 | 5713 | 5693 | 5679 | 5704 |
| Entropy Score^*^ | 1.00 | 0.99 | 0.99 | 0.88 | 0.87 | 0.87 | 0.85 |
| LRT (bootstrap-p-value)^* *^ | - | 0.00 | 0.00 | 0.00 | 0.00 | 0.00 | 0.04 |
| * Entropy Score: This value indicates how distinctly the dyads can be assigned to the classes. The closer the value.  is to 1, the better the model fit.  * * LRT: Likelihood ratio test for the model l with C classes vs. model with C-1 classes. | | | | | | | |

Supplementary table 6 Replication LCA (COMPAS data): Characteristics of 6-class model based on most likely class membership

|  | **Classes** | | | | | |
| --- | --- | --- | --- | --- | --- | --- |
|  | **1** | **2** | **3** | **4** | **5** | **6** |
|  |  |  |  |  |  |  |
| **Class size (%)** | 31.8 % | 23.1 | 14.2 | 11.2 | 12.4 | 7.4 |
| **Informal caregivers, female (%)** | 66.1 | 99.9 | 71.5 | 0.0 | 99.9 | 3.1 |
| **Individuals with dementia, female (%)** | 77.2 | 0.9 | 81.2 | 98.3 | 0.0 | 99.8 |
| **Mean age informal caregivers (all Ø 64.5)** | 50.9 | 75.9 | 62.2 | 80.5 | 62.9 | 68.9 |
| **Mean age individuals with dementia (all Ø 79.7)** | 81.1 | 80.5 | 88.7 | 80.1 | 69.2 | 68.2 |
| **Informal care relationship (%):** |  |  |  |  |  |  |
| Couple | 0.0 | 98.9 | 2.6 | 99.9 | 98.7 | 94.1 |
| Adult-Child | 92.5 | 0.0 | 82.7 | 0.1 | 0.1 | 0.2 |
| Other/Non-kinship | 7.5 | 1.0 | 14.7 | 0 | 1.1 | 5.7 |
| **Living together (%)** | 9.9 | 98.7 | 7.0 | 90.4 | 96.6 | 92.6 |
| **Working informal caregivers (%)** | 86.8 | 8.0 | 25.0 | 2.0 | 30.7 | 29.5 |

Supplementary table 7 Original LCA (DemNet-D data): Characteristics of 6-class model based on most likely class membership

|  | **Classes** | | | | | |
| --- | --- | --- | --- | --- | --- | --- |
|  | **1** | **2** | **3** | **4** | **5** | **6** |
| **Label** | **Adult-child - parent**  **relationship & younger informal caregiver** | **Adult-child-parent relationship & middle aged informal caregiver** | **Non-family relationship & younger informal caregiver** | **Couple & male informal caregiver of older age** | **Couple & female informal caregiver of older age** | **Couple & younger informal caregiver** |
| **Class size (%)** | 22.9 | 17.1 | 8.8 | 14.0 | 31.4 | 5.8 |
| **Informal caregivers, female (%)** | 78.6 | 86.8 | 79.5 | 0.9 | 100 | 63.9 |
| **Individuals living with dementia, female (%)** | 87.0 | 87.1 | 78.0 | 99.9 | 0.3 | 42.6 |
| **Mean age informal caregivers** | 50.8 | 60.1 | 51.9 | 78.4 | 73.8 | 57.6 |
| **Mean age individuals living with dementia** | 79.5 | 87.2 | 81.8 | 77.2 | 78.7 | 64.1 |
| **Informal care relationship (%):** |  |  |  |  |  |  |
| Couple | 0.7 | 0.6 | 0.0 | 99.9 | 99.9 | 93.1 |
| Adult-Child | 94.7 | 93.7 | 46.3 | 0.1 | 0.1 | 6.9 |
| Other/Non-kinship | 4.6 | 5.7 | 53.7 | 0.0 | 0.0 | 0.0 |
| **Living situation (%):** |  |  |  |  |  |  |
| Living together | 17.1 | 40.0 | 6.1 | 99.9 | 98.8 | 96.5 |
| Living nearby | 47.9 | 46.1 | 36.5 | 0.1 | 1.2 | 3.5 |
| Other/further away | 35.0 | 13.9 | 57.4 | 0.0 | 0.0 | 0.0 |
| **Occupation informal caregivers (%):** |  |  |  |  |  |  |
| No | 19.7 | 67.1 | 16.8 | 95.9 | 99.3 | 24.9 |
| Part time | 42.5 | 28.1 | 41.3 | 4.1 | 0.7 |  |
| Full time | 37.8 | 4.8 | 41.9 | 0.0 | 0.0 | 31.5 |

Supplementary table 8 Replication LCA (COMPAS data): Association of classes and use of information sources and health care services based on most likely class membership

| **Distal**  **outcomes** | **Classes** | | | | | | **p-value (Wald-Test)** |
| --- | --- | --- | --- | --- | --- | --- | --- |
|  | **1** | **2** | **3** | **4** | **5** | **6** |  |
| Class size (%) | *31.8* | *23.1* | *14.2* | *11.2* | *12.4* | *7.4* |  |
| Information source: Medical | 60.5 % | 58.2 % | 34.0 % | 71.0 % | 54.5 % | 79.0 % | **0.0064** |
| Information source: Nursing | 50.1 % | 43.6 % | 47.7 % | 31.8 % | 48.1 % | 73.6 % | 0.14 (n.s.) |
| Information source: Civil Society | 31.3 % | 30.3 % | 24.8 % | 34.5 % | 64.5 % | 58.1 % | **0.001** |
| Health care service: Medical | 94.6 % | 85.3 % | 90.3 % | 89.7 % | 93.4 % | 92.8 % | 0.46 (n.s.) |
| Health care service: Therapeutic | 27.1 % | 34.9 % | 25.4 % | 26.5 % | 34.2 % | 39.8 % | 0.66 (n.s.) |
| Health care service: Nursing | 82.4 % | 64.7 % | 96.0 % | 58.6 % | 54.1 % | 56.4 % | **<0.001** |
| n.s.=not significant (p-value less than 0.05 is statistically significant) | | | | | | | |

Supplementary table 9 Replication LCA (COMPAS data): Association of classes and Qol-AD, CarerQoL, GHQ-12 and CANE based on most likely class membership

| **Distal**  **outcomes** | **Classes** | | | | | | **p-value (Wald-Test)** |
| --- | --- | --- | --- | --- | --- | --- | --- |
|  | **1** | **2** | **3** | **4** | **5** | **6** |  |
| Class size (%) | *31.8* | *23.1* | *14.2* | *11.2* | *12.4* | *7.4* |  |
| QoL-AD (3-52; higher score=better QoL) | 29.5 | 31.2 | 28.3 | 32 | 31.1 | 32.6 | **<0.001** |
| CarerQoL (in %, reference category “yes”)  *Satisfaction performing care duties*  *Problems with person with dementia*  *Problems with own mental health*  *Problems with own physical health*  *Problems to combine daily activities*  *Financial problems with care duties*  *Receiving support from family/friends etc.* | 86.6  54.5  36.5  35.8  49.1  7.6  85.8 | 91.4  72.8  67.5  59.8  55.9  15.5  71.5 | 94.6  57.7  30.9  35.7  46.4  5.6  84.0 | 94.0  64.0  30.9  41.3  30.8  1.9  78.3 | 85.4  68.2  62.5  58.0  53.3  21.8  57.3 | 87.5  73.8  53.8  38.7  48.6  29.1  68.7 | 0.14 (n.s.)  **0.048**  **<0.001**  **0.002**  0.22 (n.s.)  0.33 (n.s.)  **<0.001** |
| GHQ-12 (0-36; higher score=worse health) | 11.8 | 14.7 | 10.4 | 11.9 | 14.7 | 13.3 | **<0.001** |
| CANE (0-26; higher score=more unmet needs) | 1.9 | 1.1 | 1.8 | 1.3 | 1.2 | 1.6 | **0.031** |
| n.s.=not significant (p-value less than 0.05 is statistically significant) | | | | | | | |

Supplementary table 10 Original LCA (DemNet-D data): Association of classes and use of information sources and health care services based on most likely class membership

| **Distal**  **outcomes** | **Classes** | | | | | | **p-value**  **(Wald-Test)** |
| --- | --- | --- | --- | --- | --- | --- | --- |
|  | **1** | **2** | **3** | **4** | **5** | **6** |  |
| *Class size (in %)* | *22.9* | *17.1* | *8.8* | *14.0* | *31.4* | *5.8* |  |
| Information source use: Informal (%) | 86.5 | 65.8 | 74.2 | 62.4 | 68.4 | 57.6 | **0.01** |
| Information source use: Medical (%) | 66.9 | 51.3 | 55.1 | 68.1 | 73.6 | 58.8 | **0.016** |
| Information source use: Nursing care (%) | 57.5 | 66.8 | 57.8 | 44.8 | 51.0 | 55.3 | 0.10 (n.s.) |
| Information source use: Civil society (%) | 29.8 | 32.3 | 28.5 | 21.8 | 40.4 | 41.8 | 0.068 (n.s.) |
|  |  |  |  |  |  |  |  |
| Health care service use: Medical (%) | 95.2 | 89.4 | 75.2 | 96.2 | 93.2 | 93.1 | **0.005** |
| Health care service use: Therapeutical (%) | 23.7 | 20.5 | 35.3 | 28.7 | 36.3 | 49.1 | **0.018** |
| Health care service use: Nursing care (%) | 58.3 | 80.6 | 90.0 | 43.6 | 47.5 | 44.2 | **<0.001** |
| Health care service use: Support services (%) | 31.7 | 26.4 | 48.6 | 37.6 | 30.6 | 14.5 | 0.067 (n.s.) |
| n.s.=not significant (p-value less than 0.05 is statistically significant) | | | | | | | |

Supplementary table 11 Original LCA (DemNet-D data): Association of classes and Qol-AD, social inclusion, caregiver burden, caregiver health and stability of care arrangement based on most likely class outcome

| **Distal**  **outcomes** | **Classes** | | | | | | **p-value**  **(Wald-Test)** |
| --- | --- | --- | --- | --- | --- | --- | --- |
|  | **1** | **2** | **3** | **4** | **5** | **6** |  |
| Class size (in %) | 22.9 | 17.1 | 8.8 | 14.0 | 31.4 | 5.8 |  |
| Quality of life of individuals living with dementia (QoL-AD, 3-52)* | 28.8 | 26.2 | 27.2 | 30.1 | 29.6 | 29.7 | < 0.001 |
| Social inclusion of individuals living with dementia (SACA, 1-32) | 24.1 | 24.2 | 25.4 | 23.2 | 24.9 | 24.7 | 0.23 |
| Subjective caregiver burden due to… (BIZA-D PV) |  |  |  |  |  |  |  |
| cognitive losses (0-16) | 7.7** (0.46) | 9.6 (0.55) | 3.7 (0.74) | 7.5 (0.36) | 8.7 (0.35) | 9.4 (0.81) | < 0.001 |
| aggression and resistance (0-20) | 5.3 (0.44) | 7.3 (0.70) | 2.7 (0.59) | 5.5 (0.63) | 6.8 (0.42 | 7.2 (1.04) | < 0.001 |
| personal constraints/health (0-20) | 6.7 (0.58) | 10.5 (0.70) | 6.6 (1.14) | 8.9 (0.42) | 7.2 (0.63) | 10.7 (1.07) | < 0.001 |
| lack of social recognition (0-24) | 7.1 (0.48) | 9.4 (0.61) | 8.6 (1.06) | 6.0 (0.68) | 7.0 (0.42) | 11.1 (1.02) | < 0.001 |
| Health status informal caregiver (EQ VAS, 0-100) | 75.2 | 65.1 | 74.9 | 61.7 | 61.0 | 62.4 | < 0.001 |
| Stability of care arrangement (0-3) | 2.0 | 2.1 | 2.4 | 2.2 | 2.3 | 2.0 | 0.016 |
| * The ranges of the scales used are listed in brackets following the names of the corresponding instruments. The maximum scores are underlined. Interpretation: Qol-AD: The higher the value, the better the quality of life; SACA: The higher the value, the better the social inclusion; BIZA-D subscales: The higher the values, the higher the burden; EQ VAS: The higher the value, the better the health status; Stability of care arrangement: The higher the value, the more stable the care arrangement.  ** Class values presented here are mean values. In parentheses: Standard deviation (SD). | | | | | | | |

1. The value of 8.2 corresponds to the national average. This means that for every employee of an outpatient care service, there are on average 8.2 persons with care needs. For the present study, the district-specific value was calculated for each dyad (based on the postcode of the person with dementia). Districts in which there are 8.2 or fewer persons in need of care for every professional outpatient carer are categorised as districts with above-average outpatient care quota. Districts in which one outpatient caregiver cares for more than 8.2 persons in need of care are categorised as districts with below-average outpatient care quota. [↑](#footnote-ref-1)
2. The value of 63.9 corresponds to the national average. This means that there are 63.9 general practitioners/psychotherapists per 100,000 inhabitants at district level. Districts with 63.9 or more doctors/psychotherapists are categorised as "above-average". Districts with less than 63.9 are categorised as "below-average". [↑](#footnote-ref-2)
